# From Machining Chips to Raw Material for Powder Metallurgy—A Review

**DOI:** 10.3390/ma14185432

**Published:** 2021-09-20

**Authors:** Catarina Duarte Batista, Adriana André Martins das Neves de Pinho Fernandes, Maria Teresa Freire Vieira, Omid Emadinia

**Affiliations:** 1 CDRSP—Centre for Rapid and Sustainable Product Development, Polytechnic Institute of Leiria, Rua General Norton de Matos, Apartado 4133, 2411-901 Leiria, Portugal; catarina.baptista@ipleiria.pt; 2 CEMMPRE—Centre for Mechanical Engineering, Materials and Processes, University of Coimbra, Pinhal de Marrocos, 3030-788 Coimbra, Portugal; teresa.vieira@dem.uc.pt; 3 Department of Metallurgical and Materials Engineering, Faculty of Engineering, University of Porto, 4200-465 Porto, Portugal; up201705636@edu.fe.up.pt; 4 Department/Faculty, LAETA/INEGI—Institute of Science and Innovation in Mechanical and Industrial Engineering, 4200-465 Porto, Portugal

**Keywords:** metal chips, milling, powder characteristics, sintering, additive manufacturing

## Abstract

Chips are obtained by subtractive processes such as machining workpieces and until recently considered as waste. However, in recent years they are shown to have great potential as sustainable raw materials for powder technologies. Powder production from metal chips, through the application of solid-state processes, seems to be an alternative to conventional atomization from liquid cooled with different fluids. However, chip material and processing have an essential role in the characteristics of powder particles, such as particle size, shape, size distribution and structure (4S’s), which are essential parameters that must be considered having in mind the powder process and the metallurgy applications. Moreover, different approaches refereed in the application of this new “powder process” are highlighted. The goal is to show how the actual research has been transforming subtractive processes from a contributor of wastes to clean technologies.

## 1. Introduction

The evolution of technology and increase in population have directed human beings to consumerism, facing exponential industrial growth with environmental impacts, such as consumption of minerals, generation of energy and production of wastes, highly seen in the steel industry. Thus, circular economy is highlighted regarding recycling or adding value to chips through the definition of new applications [1]. Hence, different studies evaluated the reuse of metal chips in various applications, e.g., the replacement of sands by metal chips in concrete production [2], the production of porous structures by sintering powders mixed with metal chips [3] or the use of steel chips as reinforcement in metal matrices [4,5]. However, the success of these applications depends on the state of interactions of chips with the matrix, e.g., the oxidation of chips in concrete may occur; the bonding between matrix and chips or between chips–chips is essential for the strength of densified product. Regarding the success of using metal chips as new resources, one study highlighted the effect of extrusion temperature and rate of extruding on the densification of product, though authors reported that the mechanical properties of the extruded product were poorer than that of the commercial material, that shortcoming was attributed to the porosity formed in the extruded chips [6].

Other application for metal chips can involve powder production by milling. This approach seems noticeable since the use of these materials appears sustainable [7,8]. Moreover, metal chips are available as major wastes in the machining industry and the use of these materials for powder production is in the scope of Goal 12 of the 2030 Agenda (Transforming our World: the 2030 Agenda for Sustainable Development). The transformation of metal chips into powders requires performing intense milling, this production consists of inducing fracture in chips by milling in conventional conditions with and without a cryogenic environment. These treatments can introduce different degrees of metastability (such as nanostructures) in the chips material by severe plastic deformation. The current study reviews the state-of-the-art methods for powder production from metal chips considering milling conditions and powder characteristics, mainly the 4S’s, such as particle size, particle size distribution, particle shape and the structure of particles, as well as the potential applications for these powders.

## 2. Materials of Chips

The metal chips used for recycling can involve industrial residues [9] or as machined fresh type [10], resulting from operations such as turning [11,12], roughing and finishing [13] obtained by low speed machining [14,15] or a high speed process [13]. Materials studied so far involve aluminum alloys, stainless steel, tool steel, titanium alloy, tin alloy and nickel alloy, with different initial sizes.

## 3. Powder Production

Milling techniques can include: (1) attritor ball milling that can be categorized as dry grinding or wet grinding, using regular speed (to 400 rpm) or high speed (400–1800 rpm) attritors [10,16]; (2) roll milling [17]; (3) planetary type [7,13]; (4) disc milling [10,18,19]. Powder production mostly involves fracturing cleaned and dried chips/swarf; meanwhile, cold welding between fragments may occur; however, this joining depends on the energy and conditions of milling. Chips face plastic deformation and consequently fracture. The fragmentation occurs by compaction or impaction accompanied or not with friction depending on the milling technique and procedure. Moreover, particle fragments can be associated with grain refinement, possible to reach nanocrystallinity through high energy milling [20].

Regarding mechanical milling processes, such as the planetary technique, it is important to consider the processing parameters and conditions. Milling energy is influenced by milling time and revolutions per minute (rpm), even ball to powder ratio (BPR). Other conditions, such as the milling atmosphere, the use of process control agent (PCA), and the temperature of milling, are significant too. These conditions affect the evolution of the 4S’s of the milled product [21]. Moreover, the characteristics of the milling, such as the material and size of the milling jar and that of the balls, seem to be effective; one study showed that coarser particles were the result of balls with 20 mm diameter than balls of 6 mm [22].

Though the application of cryogenic ambient during milling seems to be effective for powder fragmentation, influencing ductile to brittle fracture temperature transition, some authors reported that disc milling was more productive than ball milling or cryogenic milling/grinding system, taking into account the time of milling for transferring all chips into powders [18].

The jet milling technique was also applied for the powder production from bronze chips [23]. This technique implemented a jet flux for impacting chips on a hard target by which impaction and attrition, rather than wear, were responsible for fragmentation. The impact angle and the distance between the nozzle and target were effective parameters. It revealed that fragmentation by ball milling required larger time in comparison with jet milling by which a faster fragmentation and a greater efficiency were achieved; however, the effect of jet milling was pronounced in the first cycle attributed to the presence of cracks and defects caused by the machining process [23].

Fragmentation is greatly influenced by the type of milling process applied on chips, a higher efficiency of powder production was highlighted for the mill shaker technique than the planetary milling, achieving a faster size refinement was attributed to the milling energy induced by the shaking process [24]. Moreover, the collision energy also depends on the milling technique. Some authors attributed the efficiency of a high fragmentation to impact energy implemented by disc milling in comparison with cryogenic mill and ball miller [18].

In addition, the application of PCA is important; it is usually used to avoid adhesion between particles to balls or to prevent cold welding between particles in order to dominant fracturing; PCA can define the distribution size and morphology of the milled particles. Methanol and stearic acid (SA) are common PCAs. One study presented the production of a d_50_ of 100 µm to 325 µm; however, the finest particles (d_50_ of ~140 µm) were produced by introducing 0.5 wt.% SA to the chips before milling [8].

Regarding the milling atmosphere, the most common is Argon gas, which is very useful to avoid oxidation of metal particles, as contaminations, during milling [20]. However, other strategies can be selected for that purpose, such as adding PCA, e.g., a toluene environment [25].

Regarding the effect of BPR, the application of more balls increases collisions with particles increasing fragmentation. Therefore, the application of a higher BPR can increase the efficiency of milling [25]. Thus, it would be interesting to evaluate the effect of BPR on fragmentation vs. the influence of milling time for future objectives as a solution for reducing energy consumption.

## 4. Characterization of Powder Particles Produced from Metal Chips

### 4.1. Particle Size

Particle size analysis determines the progress of fragmentation during mechanical milling chips for powder production. This analysis can be performed by several techniques: sieving that is reported in wt.%, image analysis acquired by transmitting optical microscope (OM) or stereoscopic macroscope (SM) or scanning electron microscope (SEM). Other techniques include static light scattering (SLS), laser particle size analyzer (LPSA) or laser diffraction (LD) particle size analyzer (PSA).

The evolution of particle size is governed by the fragmentation of metal chips and is dependent on the materials characteristics and processing conditions as well. Regarding the influence of material, some authors showed the production of particles in dissimilar sizes from similar milling of different chips; that difference was attributed to the primary conditions of the chips used for milling [17]. Table 1 shows the function of the material chips, the different studies performed during the last decennia concerning particle size and the milling process.

Some authors revealed the success of applying a pre-heat treatment on a Ti alloy, heating in an H_2_-Ar mixture atmosphere, on decreasing particle size, the production of fine particles was attributed to the formation of brittle TiH_2_ compound during the pre-heat treatment step [26]. Another study evaluated the effect of chemical composition on fragmentation, the application of similar milling conditions on two steels, an extra low carbon steel and a low carbon type, the former type faced with agglomeration formation caused by cold welding. Moreover, same authors revealed that the larger the BPR ratio, the finer the particle size.

Regarding the achievement of smaller particle size, some studies performed showing that the fragmentation can increase in the presence of a harder phase mixed with the matrix during milling; some authors proved this concept by milling Ti_6_Al_4_V chips mixed with 10 wt.% of alumina particles, showing a sharp reduction of particle size [27].

**Figure 1:**
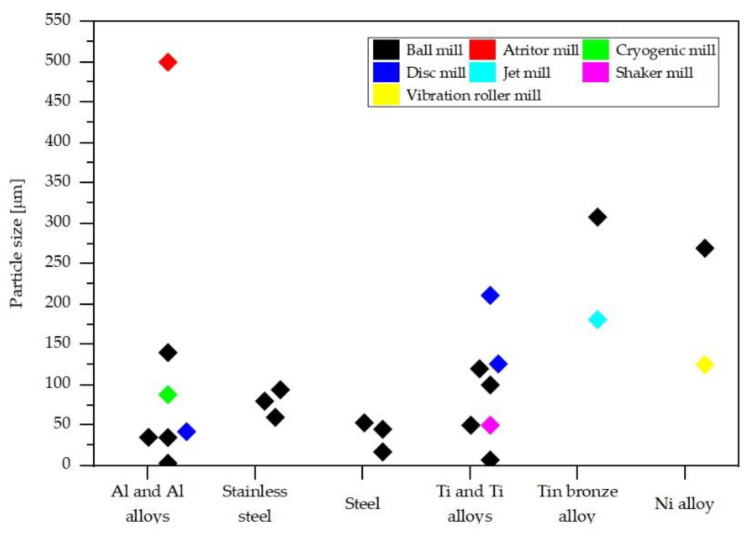
Illustration of particle size mentioned in Table 1.

Milling time also influences fragmentation and particle size formation, e.g., an increase from 3 h to 5 h milling increased yield fragmentation [25]. Fragmentation continues by prolonging milling time though agglomeration of fine particles and formation of clusters can appear, e.g., particles of ~1 μm, in clusters, formed within 50 h ball milling of Ti_6_Al_4_V particles [24]. Figure 1 illustrates the particle size of recycled powders in different alloys influenced by processing methodology and conditions.

### 4.2. Particle Size Distribution

The particle size distribution (PSD) can be affected by the presence of additives, such as PCA and even reinforcements (e.g., niobium carbide (NbC), vanadium carbide (VC), silicon carbide (SiC) or Titanium carbide (TiC)), milling time or by the technique chosen for milling. Regarding the use of SA as a PCA, the procedure of addition is very important. Table 2 shows the function of the material chips and the different studies performed during the last decennia concerning particle size distribution function of milling conditions (Table 1). Some authors revealed the production of finer particles, d_50_ of almost 100 µm, by the addition of SA in the beginning of the milling process [8]. As regards the fragmentation in the presence of reinforcement, an addition of a 3% NbC resulted in narrowing particle size distribution [28].

A comparative study between jet milling and ball milling revealed a larger peak broadening for the PSD of particles obtained by ball milling attributed to the hardening of particles that occurred during the ball milling process [23].

### 4.3. Particle Shape

**Figure 2:**
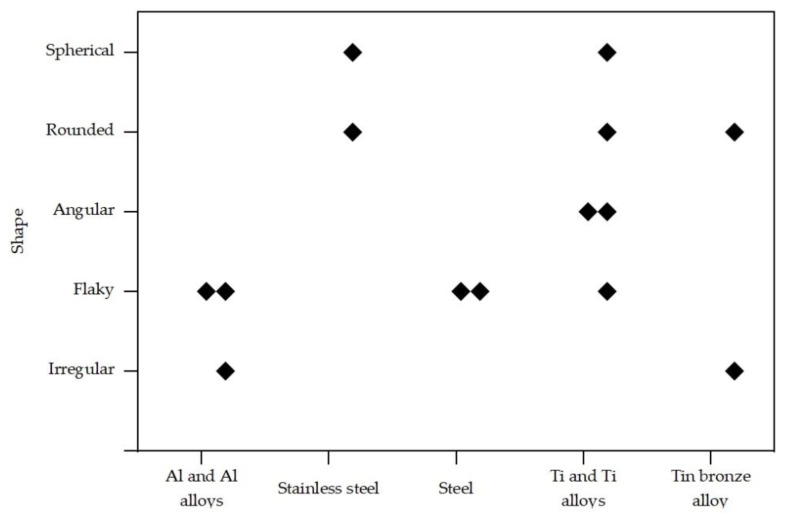
Illustration of particle shape mentioned in Table 3 (diamond inserts in this graph represent studies discussed in this review article, as cited in Table 3).

Table 3 and Figure 2 summarize the different research works about the particle shape function of material and the milling process (Table 1). The morphology of the milled particles is strongly influenced by the milling time, i.e., during the initial steps a flake-like shape is obtained, this shape changes to spherical particles by prolonging milling time by which particles are repeatedly cold welded and fractured [24]. Some authors reported the production of H13 particles with metallic appearance of an aspect ratio close to one by prolonging milling time [13].

Moreover, the powder shape is influenced by the milling technique, the production of angular and agglomerate-free particles was remarked by the performance of shaker milling that was independent on milling time [24]. The diameter of the balls used in mechanical milling also has an influence on the morphology of the powder particles. Applying a similar BPR, large balls (diameter = 20 mm) efficiently break up chips to coarse powder particles while small balls (diameter = 6 mm) effectively modify the powder morphology to near-spherical [22]. Some authors highlighted the effectiveness of ball size on the morphology of milled particles, i.e., a two-stage ball milling starting by large balls and ended with small balls, φ = 6 mm and 20 mm, resulted in the production of near-spherical particles [22,34].

In addition, other authors showed that the use of PCA, such as SA, influenced the particle shape, depending on the milling time in the presence of SA [8].

Regarding particle shape applied in powder metallurgy, ISO 3252:2019 provides complete information, defining acicular particles to spheroidal type, defined by microscopic observations. However, microscopic images can be evaluated quantitatively, defining aspect ratio (proportion of the longest diameter to the shortest aspect of one particle). Nonetheless, a recent study applied the plasma spheroidization technique on milled particles in order to accomplish the aspect ratio of one so that spherical shaped particles could be achieved; however, the shape factor was dependent on the powder feeding rate during the spheroidization process [16].

### 4.4. Particle Structure

Table 4 shows the structure of milled chips for different materials. Some researchers illustrated the presence of sub-micrometric to nanometric grain sizes as well as a martensitic structure H13, (AISI) tool steel, chips produced by a machining process in air [35]. A recent study based on SEM, X-ray diffraction (XRD), Transmission Electron Microscopy (TEM) and Electron Backscatter Diffraction (EBSD) obtained by TEM (t-EBSD), revealed that the microstructure inside the adiabatic shear band, consisting of severe deformation, is composed of ultrafine and nanocrystalline grains, adjacent areas included thin martensite laths with high dislocation density and nanocrystalline grains as well [36]. The target was to highlight the effectiveness of milling metal chips for powder production. Nevertheless, the creation of nanostructured powders was also desired. Some authors also reported the production of nanometric martensitic grains through the introduction of extremely high strain rate into austenitic stainless steel chips [7]. Moreover, other researchers showed that the application of shaker milling, that is a higher energetic process than ball milling, revealed a faster rate to achieve nanocrystallinity—10 h of shaking was equivalent to 40 h of mill in a planetary milling [24]. Some authors mentioned the effectiveness of milling speed on attaining particle size, a non-uniform particle size distribution (211 µm produced at 1200 rpm for 24 min) without any change in the structure, the crystallite size decreased by an increase in milling speed and not milling time [29]. Considering the milling time, some authors attributed the increase in grain size to the heat effect caused by long milling time [8]. Nonetheless, the crystallite analyses in these studies were performed by X-ray diffraction (XRD), considering the width of peaks, EBSD or TEM. Magnetic analysis can confirm if austenite microstructure has transformed into martensite; some authors showed that annealing at 700 °C, for 1 h, reduced the strain but the microstructure still remained with a significant proportion of martensite [7]. Regarding the influence of the milling process, for instance in TiAlV alloy, some authors showed that the peak broadening in XRD was higher in powders resulting from shaker milling than ball milling [24]. 

The efficiency of shake or jet millings can exceed ball milling due to a higher energy inherent to the technique. Moreover, disc milling shows a similar trend. Regarding milling conditions, an increase in milling time leads rise yield fragmentation. However, agglomeration of fine particles and formation of clusters can happen, caused by a cold welding effect. Similar behavior can be achieved by the application of the highest ball number. In what concerns temperature, a cryogenic ambient during milling also means high powder fragmentation for materials that promote a ductile to brittle fracture. The use of controlling agents such as stearic acid or toluene also influences the fragmentation. The use of a controlled atmosphere such as argon can avoid oxidation and contamination of milled particles. Moreover, the presence of additives or milling time affect powder characteristics, in particular particle size distribution. Regarding particle shape, the diameter of the balls appears to have the strongest effect on reaching near spherical powder particles. Moreover, an increment in milling time leads to a modification of shape factor to high values. Nevertheless, shaker milling lets angular shapes and non-agglomerate structures, independently of time. Concerning particle structure, shaker milling reveals a fast rate to achieve nanocrystallinity over planetary milling. However, crystallite size can decrease by increasing the milling speed and not milling time. The presence of a hard phase mixed with the matrix as reinforcement increases the chips fragmentation, promoting particle size reduction. Moreover, the addition of hard reinforcement encourages the reduction of powder crystallite size. The phase transformation from austenite to martensite phase can occur, particularly in austenitic stainless steel, due to intensive plastic deformation.

## 5. Recycling Chips for Powder Metallurgy Applications

The recycled powder particles obtained from metal chips recovery are noteworthy for powder metallurgy applications, conventional and advanced processes, such as pressing and sintering, hot isostatic pressing (HIP) or additive manufacturing (AM), respectively, including direct energy deposition (DED) or selective laser melting (SLM) Table 5 shows the densification processes applied on powder milled from different materials.

Some researchers studied compacted disks of Al powder using recycled chips, chemically cleaned prior to milling to reduce oxide on surfaces, attaining a green density of 80%; these authors attributed the lack of strength of sintered powders to the weak densification [10]. Other study applied hot isostatic pressing (HIP) to increase the density of compacted and sintered Ti_6_Al_4_V powder, but the densification of commercial powders was still higher than powders produced by milling [32]. With regards to the densification of recycled powders through conventional techniques, some authors suggested the production of porous bearings or even high-density P/M structural components, using recycled tin bronze powder [23].

Metal chips were applied also for producing metal matrix powder composites. Taking into account the milling time, increase in particle size by extending milling time caused by cold welding, some authors overcame that shortcoming in the production of a duplex stainless steel composite powder mixed with vanadium carbide (VC) particles [15]. This study also presented that: the increase in the reinforcement concentration results in the reduction of average particle size. However, a similar study shows that the hardness of the sintered composite was smaller than the as-received alloy, and the failure was attributed to the porosity of sintered composite [37]. Other researchers also exhibited a significant effect on the fragmentation of particles in the presence of reinforcements, d = 80 µm in Ti_6_Al_4_V powder and 4 µm in Ti_6_Al_4_V-10 wt.% nanoalumina [27]. The same study revealed a great reduction in the crystallite size of nanocomposite powder in comparison with non-reinforced, respectively, 15 nm and 90 nm. The hardness of the nanocomposite increased 168%. Thus, grain coarsening did not occur in the annealed nanocomposite powders, at 600 °C for 1 h, whereas the milled-annealed Ti_6_Al_4_V powders showed significant grain coarsening [27]. Other researchers evaluated the recycling of a duplex stainless steel in the presence of 3% of NbC by milling, the transformation of austenite to martensite occurred, induced by severe plastic deformation though that transformation reduced in the presence of NbC [28]. Some researchers produced in situ TiC reinforced Ti composite powder by adding graphite to Ti chips; these authors showed that the graphite behaved as an inhibitor for fragmentation as well as oxidation [31].

Additive manufacturing, despite the high costs involved, has been acquiring significant importance due to its sustainability and the reduction of waste [34], obtained by skipping subtractive machining processes to achieve the final product. Other advantages from this technique are the possibility to make complex geometries, high production and low cost of transport and storage. In this process, mechanical milling is an alternative for gas atomization (GA) [34], the most common technique to produce powder with a shape factor close to 1 and controlled particle size distribution. GA is not the best option since it consumes a lot of energy, resulting in high costs and limited alloys composition for production by atomization [34,38]. Thus, powders for AM should have appropriate 4S’s, depending on technique, e.g., d_90_ of 40–140 µm for direct laser deposition (DLD) or d_90_ of 20–50 μm for selective laser melting (SLM) and d_90_ of 10 μm for fused filament fabrication (FFF) [39,40]. Some authors developed an optimized procedure to produce powders appropriate for AM, a two-stage milling approach, i.e., balls with a diameter of 20 mm were used to fracture the steel chips and then balls with a diameter of 6 mm allowed the powder to acquire a shape factor close to 1 with a smoother surface feature. Moreover, as the milling time increased, less flattened are the particles [22]. However, a recent study performed atomization of milled particles to produce spherical shaped powders suitable for AM processing, such as DED and SLM technologies, reaching relative densities of 99% [16].

### Mechanical Properties

The ball milling can induce plastic deformation as well as accumulation of internal strain, leading to the grain refinement of powder structures. This effect can induce strengthening, e.g., some authors reported the increase in hardness (850 HV) for stainless steel recycled powder (100 h of milling), the initial chips a hardness of 374 HV. This influence is attributed to the formation of a nanosized martensite phase [7]. A similar trend was observed in other metals, affected by milling time as well [8,9,11,22,23,24,32].

Regarding the mechanical properties of materials after densification, Table 6 revealed that mechanical properties, such as hardness or strength, depend on material composition and processing, appearing additive manufacturing is more promising.

The reduction in mechanical properties, such as strength and hardness, of recycled powders is attributed to efficiency of densification [8,10,23,32].

## 6. Conclusions and Future Perspectives

From machining work parts is feasible for a significant number of metallic material chips (aluminum alloys, carbon steels, stainless steels, tool steels, Ti-Al alloys, superalloys) to produce powder through milling techniques. In general, these new “raw materials” take the advantage of severe plastic deformation to proceed with the fragmentation of chips. Moreover, the addition of other materials (i.e., nanoceramics) could also promote the fragmentation of ductile metallic alloys.

Depending on the pristine material, powder milling of chips have a main role in the final powder characteristics (4S’s). The new powder particles resulting from the milling of metallic chips can be applied mainly to powder metallurgy technology approaches, from conventional processes (subtractive and replicative) to additive manufacturing. This review highlights the role of metallic chips in manufacturing, and it enhances that the production of wastes is not a prerogative of subtractive process, it could be essential to additive manufacturing to guarantee its sustainability.

Regarding future perspectives, it can involve the transformation of chips in outstanding powder raw material with characteristics not available in atomized powder particles. This target will be attained by a detailed study of microstructures function of waste material and processing, milling conditions and the nanoreinforced additions. Particular attention will be dedicated to the technologies selected for 3D object production.

## Figures and Tables

**Table 1:** Milling of different material chips.

	Input Material	Conditions	Results	Analysis Method	Reference
Al and Al alloys	Aluminum in max. 6 mm length	Cut milling chips to 4–2 mm, attritor milling (300 rpm, 20 mm φ balls BPR of 10:1) in air	<500 μm	Sieved	[10]
	AlSi_5_Cu_2_ aluminum alloy with 250 µm, heat treated	High energy ball mil (200 rpm, BPR of 10:1) in argon, with 1 wt.% stearic acid and with SiC particles (10, 20 wt.%) for 40 h	3 µm	SEM	[11]
	AA7075 (10 mm × 2 mm × 0.5 mm) after cut by double roller	High energy planetary ball milling (400 rpm, carbide tungsten vials, BPR of 30:1) in argon and with 2% methanol for 0.5 to 10 h	5 h: d_50_ = 75 µm 10 h: d_50_ = 35 µm	SEM LPSA	[12]
	AA6013-T6 in 4–10 mm	(1) Cryogenic milling (25 mm φ stainless steel ball, nitrogen cooling) stages for 24 min (2) Disc milling (1500 rpm) for 4 min (3) Ball milling (550 rpm, 25 mm φ stainless steel ball) stages of 40 min	(1) Cryogenic mill: d_50_ = 88 µm (2) Disc mill: d_50_ = 42 µm (3) Ball mill: not mentioned	LD	[18]
	AA2024 20 mm × 9 mm × 0.1 mm	Planetary ball milling (300 rpm, 16 mm φ stainless steel balls, BPR of 10:1) in air in different regimes with and without SA for 100 min	d_50_ of ~140 µm by an optimized regime	SEM OM SLS	[8]
	Aluminum metal swarf with 3 mm after blended	Ball milling (370 rpm, 10 mm φ 36 zirconia balls) with and without PCA	d_50_ = 34.829 µm at least 7 h of milling	LD	[9]
Stainless Steel	Stainless steel in 2–4 mm discontinuous, non-stringy and C-shape	Planetary ball milling (400 rpm, 20 mm φ hardened chromium steel balls, BPR of 10:1) in argon for 25, 50, 100 h	50 h: 300 µm 100 h: 60 µm	SEM	[7]
	UNS S31803 duplex stainless steel, thin and small chips short spiral type machining at low speed 2 mm of mean size	Planetary ball mill (350 rpm, 5 and 20 h, BPR of 15:1) in argon with and without 3% NbC for 5 and 20 h	5 h: 600 μm 20 h: 219.7 µm 5 h + 3%NbC: 150–500 µm 20 h + 3%NbC: 175.6 µm	SM SEM PSA	[28]
	UNS S31803 duplex stainless steel machining at low speed 5–15 mm	Planetary ball mill (250–350 rpm, BPR of 20:1) in argon and 0–3% VC for 10 to 50 h	25–135 µm at more aggressive conditions	SEM PSA	[5]
	AISI 304L Chips with serrations along the length 5–20 mm	Planetary ball mill (500 rpm 6 and 20 mm φ stainless steel balls, BPR of 15:1) in argon	38–150 µm by an optimized regime	Sieved	[22]
	Fe-11Cr-1.5Ni-0.2V-0.4Mo-0.1C shear-localized chips with 5–6 mm in length and 1 mm in thickness	Attritor with grinding ball (260 rpm, 7–10 mm φ balls, BPR of 10:1, 40 kg ball charge) with 0.2 wt.% stearic acid in argon for 6–12 ks	91 wt.% content of powder particles <125 µm form 12 ks	Sieved	[16]
Steel	Three different steel chips 10 mm across and 1.5 mm thickness	Rolling ball mill (231 rpm, 10 mm φ porcelain balls) for 2 h and 6 h	6 h ≥ finer particles	Sieved	[17]
	AISI H13 high speed machining chips with 0.1–0.5 mm (finishing) and 5–7 mm (roughing)	Planetary ball mill (300 and 450 rpm, 20 mm φ hardened chromium steel balls, BPR of 20:1 and 10:1) in Ar + H_2_ (5%) until 300 min	d_50_ = 53 µm at 450 rpm, 180 min BPR of 10:1	L DSEM	[13]
	Low carbon steel and extra-low carbon steel (LCS and ELCS, respectively)	High energy dual drive planetary mill (Jar:620 rpm, main shaft: 275 rpm, 10 mm φ stainless steel balls, BPR of 12:1 and 6:1) immersed in toluene	LC 3 h: 5–10 µm (12:1) and 15–20 µm (6:1) 5 h: 15–20 (6:1) ELC 5 h (12:1): 40–50 µm	SEM	[25]
Ti and Ti alloys	Ti_6_Al_4_V scrap chips in spring shape size chips with <2 cm after crush.	(1) Planetary milling (500 rpm, 20 mm φ hardened carbon steel balls, BPR of 10:1) in argon for 5, 10, 20, 30, 40 and 50 h (2) Shaker milling (613 rpm, 4 mm φ hardened carbon steel balls, BPR of 10:1) in Argon for 1, 2, 8 and 10 h.	(1) Planetary mil: 5 h: <600 µm 10 h: <400 µm 20 h: ~300 µm 30 h: ~300 µm 40 h: ~200 µm 50 h: <100 µm (2) Shaker milling 1 h: ~800 µm 2 h: <700 µm 8 h: <400 µm 10 h: <50 µm	SEM	[24]
	Ti_6_Al_4_V machining chips in spring shape	Planetary ball mill (500 rpm, 20 mm φ hardened carbon steel balls, BPR of 10:1) in argon for 5, 10, 20, 30, 40 and 50 h with and without 10 wt.% Al_2_O_3_ nanoparticles	50 h: <80 µm With Al_2_O_3_: 20 h: ~225 µm 30 h: ~6 µm 50 h: ~4 µm	SEM	[27]
	Ti_6_Al_4_V spiral machining chips, heat treated at different temperatures in H_2_-Ar	Planetary ball milling (200 rpm, BPR of 12:1, 10 mm φ ZrO_2_ balls) for 10, 30, 60 min	d_50_ = 120 µm obtained by 60 min milling of chips heat treated at 800 °C for 30 min	LD	[26]
	Ti_6_Al_4_V machining scraps in spring-like shape	Disc milling (800 to 1400 rpm) in air atmosphere for 4, 8, 12, 16, 20 and 24 min	d_50_ = 211 µm at 1200 rpm for 24 min	SEM PSA	[29]
	Ti_6_Al_4_V	High energy planetary ball mill for 3 h	≤50 µm	-	[30]
	Ti spring shape chips with 8–10 mm after cutting	Dual drive planetary mill (jar:620 rpm, main shaft:275 rpm, 8 mm φ stainless balls, BPR of 10:1) immersed under toluene for 2.5 h	5–10 µm	SEM FESEM	[31]
	Ti_6_Al_4_V 2000 µm	Vibratory disc mill (700, 800, 900, and 1000 r/min at different sieving sizes 2000, 500,300 and 200 μm)	40–212 µm	Sieving	[32]
Tin bronze alloy	Tin bronze alloy 595–841 µm	(1) Ball mill (60 rpm, tool steel balls, BPR of 20:1) in air for 4, 8, 16 and 24 h (2) Target jet mill (nozzle to target distance:8 cm, impact angle: 90°, compressed air: 6 bar) for 1, 3, 5, 7 and 10 h	(1) Ball mill: 24 h: 308 µm (2) Target jet mill: 10 h: 181 µm	SEM Sieving	[23]
Ni alloy	Alloy metal with 77% Ni chips Crusher in a hammer mill to <2.5 mm	(1) Eight-chamber continuous-discharge vibration roller mill (f = 19.5 Hz and A = 6.7 mm) in four stages (2) Batch-operated cantilever-type ball mill (71 rpm, 40, 25 and 20 mm φ balls with ratio 50:34:16, 87.5 h)	(1) Vibration mill: 51.5% of particles < 125 µm; (2) Ball mill: 63.1% of particles < 125 µm.	Sieving PSA	[33]

**Table 2:** Particle size distribution.

Input Material	Particle Size Distribution	Analysis Method	Reference
AA2024	d_50_ = 100–325 µm	SLS and SEM	[8]
Stainless steel	Reported as narrow size distribution	SEM	[7]
UNS S31803 duplex stainless steel	Without NbC: d_10_ = 65.3, d_50_ = 134.8 d_90_ = 473.3 With 3% NbC: d_10_ = 65.6, d_50_ = 115.8, d_90_ = 355.7	SEM and PSA	[28]

**Table 3:** Particle shape.

Input Material	Particle Shape	Analysis Method	Reference
AA7075	5 h milling is the critical time for morphology change, chips with segmented shape to irregular powder morphology	SEM	[12]
AA6013	Flaky and irregular shaped. Average aspect ratio of 1.25 (disc mill), 1.42 (cryogenic mill) and 1.63 (ball mill)	OM	[18]
AA2024	Flake shaped	SEM	[8]
Stainless steel	Spherical powders	SEM	[7]
AISI 304L	Rounded particles and smoother surfaces with average aspect ratio of 1.37	SEM	[22]
Fe-11Cr-1.5Ni-0.2V-0.4Mo-0.1C	Flake shape after milling and spherical (>85%) or rather spherical shaped particles after plasma spheroidization applying a feeding rate of 30 g/min	Tomography	[16]
Three different steel chips	Flake shape	-	[17]
Ti_6_Al_4_V	Planetary ball mill: 5 h: Flake shape 10 < h < 50: spherical Shaker ball mill: 2 h: flake-like 10 h: angular	SEM	[24]
Ti_6_Al_4_V	5 h: Flake shape 10 h: Equiaxed shaped particles 20 h: Large cluster (agglomerated particles) 30–50 h: decrease in clusters With 10 wt.% Al_2_O_3_: 2 h: Flake shape 10 h: Equiaxed in shape 20 h: Smaller particles	SEM	[27]
Ti_6_Al_4_V	Spring-like scraps turn into flaky powder form	SEM	[29]
Tin bronze alloy	Jet milling powder: irregular shape Ball milling powder: rounded with smooth surface	SEM	[23]

**Table 4:** Structure of milled particles.

Input Material	Initial Structure	Final Structure	Reference
AlSi5Cu2	-	Elongated subgrains in 1 µm length (without SiC), spherical subgrains of size below 50 nm (20 wt.% SiC after 40 h milling)	[11]
AA2024	Nanocrystalline structure (~ 10 nm)	Nanocrystalline structure (25–45 nm)	[8]
Stainless steel	Austenite + martensite	Martensite is dominant after 25 h milling	[7]
UNS S31803 duplex stainless steel	Ferrite + austenite	0	[28]
AISI 304L	Austenite + martensite	Transformation of primary austenitic phase into martensitic one	[22]
AISI H13	bcc martensitic phase with a fine dispersion of V_x_C_y_ Ultrafine and nanocrystalline grains ($\bar{d}$ = 85 nm)	-	[36]
Low carbon steel and extra-low carbon steel	-	Grain size of 10–20 nm	[25]
Ti_6_Al_4_V	Crystallite size of 90 nm	Ti_6_Al_4_V and Ti_6_Al_4_V + Al_2_O_3_ with crystallite sizes of 20–15 nm	[27]
Ti	-	Grain size of 10–20 nm	[31]

**Table 5:** Applications of milled particles.

Powder from Chips	Reinforcement	Densification Process	Reference
Aluminum	-	Pressing	[10]
AlSi_5_Cu_2_	SiC	Nanostructured composite powders	[11]
AA2024	-	Hot-pressing	[8]
UNS S31803 duplex stainless steel	NbC	-	[28]
UNS S31803 duplex stainless steel	VC	Metal-carbide composites	[15]
UNS S31803 duplex stainless steel	VC	Metal-carbide composite Heat treatment followed by isostatic pressing	[37]
AISI 304	-	Additive manufacturing—DED	[22]
Fe-11Cr-1.5Ni-0.2V-0.4Mo-0.1C	-	Additive Manufacturing—SLM & DED	[16]
Low and extra-low carbon steel	Nanoyttria	Pressing and sintering	[25]
Ti_6_Al_4_V	10 wt.% Al_2_O_3_	Nanocomposite powder	[27]
Ti_6_Al_4_V	-	Pressing and sinter-HIP	[26]
Ti_6_Al_4_V	-	Pressing and sinter-HIP	[32]
Ti	Graphite	TiC reinforced Ti-TiC composite powder	[31]
Tin bronze alloy	-	Pressing	[23]

**Table 6:** Mechanical properties after application of milled particles.

Material	Densification Methodology	Analysis	Results	Reference
Aluminium	Pressing	Compressive strength Brinel hardness	Green compacted with commercial powders had higher compressive strength (138 MPa) than powders from chips (120 and 135 MPa) Hardness of commercial powders (28 BHN) higher than powders from chips (20 and 23 MPa)	[10]
AA2024	Hot-pressing	Microhardness	Hardness of as-compacted material close to bulk alloy (108 HV)	[8]
UNS S31803—VC composite	Isostatic Pressing	Microhardness	Decrease in hardness for 13% less	[37]
AISI 304	Additive manufacturing—DED	Nanoindentation	Single track of powder from ball milling had 21% more hardness than single track of powder by gas atomization	[22]
Fe-11Cr-1.5Ni-0.2V-0.4Mo-0.1C	Additive Manufacturing—SLM & DED	Tensile strength Yield point Elongation	Tensile strength and yield point above standard requirements and small elongation with and without heat treating	[16]
Low and extra-low carbon steel	Pressing and sintering	Microhardness	Higher hardness in sintered powder with the addition of nanoyttria (LCS: 140.8 HV and ELCS: 87.1 HV)	[25]
Ti_6_Al_4_V	Pressing and sintering followed by HIP	Flexural strength	Sintered compacts with commercial powder had higher strength (~450 MPa) then powder from chips (~370 MPa)	[32]
Tin bronze alloy	Pressing	Green strength	Powder from jet milling (~12 MPa) had higher strength then powder from ball milling (~5 MPa).	[23]

## Data Availability

Data sharing is not applicable to this article.
